# The Anne Boleyn Illusion is a Six-Fingered Salute to Sensory Remapping

**DOI:** 10.1177/2041669516669732

**Published:** 2016-09-21

**Authors:** Roger Newport, Dominic Y. Wong, Ellen M. Howard, Eden Silver

**Affiliations:** School of Psychology, University of Nottingham, Nottingham, UK

**Keywords:** multisensory illusions, body representation, touch, remapping, public engagement

## Abstract

The Anne Boleyn Illusion exploits the somatotopic representation of touch to create the illusion of an extra digit and demonstrates the instantaneous remapping of relative touch location into body-based coordinates through visuo-tactile integration. Performed successfully on thousands, it is also a simple demonstration of the flexibility of body representations for use at public events, in schools or in the home and can be implemented anywhere by anyone with a mirror and some degree of bimanual coordination.

## Introduction

The sensation of touch on the skin is not enough, by itself, to know the precise location of that stimulus on the body surface. For that, the location of touch on a somatotopic neural map (identified purely in relative terms to other locations on the map without reference to specific locations on the body) must be remapped onto a body-based frame of reference (Badde, Röder, & Heed, 2015; Longo, Azañón, & Haggard, 2010). Furthermore, due to the nature of multisensory integration, the localization of touch can be modulated by concurrent postural, visual, and auditory sensory cues, although the precise nature of such interactions remains a topic of much debate (Noel & Wallace, 2016). The illusion described here exploits the somatotopic representation of touch to create the perception of having an additional finger. Named after Anne Boleyn, wife of Henry the VIII of England, for whom there is a common misperception of her having possessed six fingers on one hand, whereas closer inspection of the evidence suggests that she did not (Bell, 1877), the illusion described here gives the distinct impression of having six fingers, whereas, in reality of course, there are not. The illusion involves the use of a mirror to duplicate the appearance of body parts symmetrically; a technique used to good effect for at least 50 years (Driver, Maguire, & Powell, 1960; Ramachandran, Rogers-Ramachandran, & Cobb, 1995).

The full procedure is described in Supplemental Figure S1, but briefly: The participant’s view of the right hand was replaced by a reflection of the left hand using a flat mirror placed vertically between the hands. The experimenter stroked the hand seen in the mirror once along the top of each digit and then once on the table surface next to the little finger. At the same time, and in temporal synchrony, the hand hidden behind the mirror was stroked once along the top of the first four digits, then on the inner and outer surfaces of the little finger. The hypothesis was that the integration of vision and touch for the stroke on the inner surface of the finger would create a percept of being touched near the top of the finger on both hands. If the first stage of touch localization is processed according to somatotopic mapping of the skin, then the final touch on the hidden outer surface of the finger should be felt to be in a distinctly different place to, and more eccentric than, the preceding touch on the inner surface. Integration with visual feedback of a touch that is also more eccentric (on the table next to the finger) should create the sensation of touch next to the existing little finger. If, on the other hand, there is a direct point-to-point mapping between touch on the skin and location on the body, then the illusion should not be effective and touch will be perceived to be in its veridical location on the outer surface of the finger.

Investigation comprised three distinct approaches completed simultaneously: Detailed data were collected under laboratory conditions from 18 naïve adults recruited from a mixture of university students and members of the public; simple response data were collected from a random sample of children (*n* = 422) attending a major science fair; and the general efficacy of the illusion was observed in 3,500 members of the public at the same fair.

**Figure 1.**
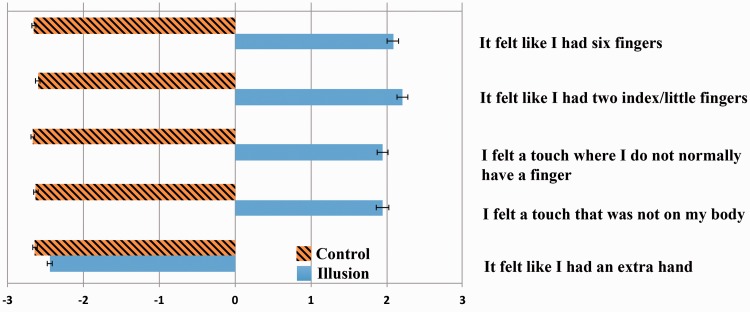
Data were pooled across both finger versions of the illusion from the laboratory experiment. For the four experimental statements, all significance values (uncorrected *t*-tests) were less than *t*(17) = 17.5, *p* < .0000000001. The control question (bottom) was not rated positively by any participant.

For the laboratory experiment (in 12 males, 6 females, aged 19 to 32; mean 23), the illusion procedure was compared with a control block in which the little finger was stroked twice and seen to be stroked twice in the mirror reflection. The routine was conducted twice within each block, and two additional blocks involved a slight modification for which the index finger was the illusory target instead of the little finger (stroking the unseen index finger on the inside and outside while stroking the table between the index finger and thumb for the reflected hand). No significant differences were observed between index and little finger illusions. Six fingers were reported on 67 out of 72 illusion trials (only five fingers for all control trials). Participants also reported that it felt as though they had two index or little fingers (further data are given in Figure 1).

Of 422 children (64% female) who received the little finger version of the illusion in non-laboratory conditions, 73% reported feeling a sixth little finger after one presentation, 85% after two, and only 11% reported no sensations of extra fingers or weirdness at all (the other 4% reported a sense of intense “weirdness” but no extra finger). These data mirrored the general observation across all 3,500 participants that the effect is highly universal. While formal data were not collected from this large sample, outcomes were easily monitored (below) with approximately a 90% success rate.

Such is the bidirectional nature of public engagement that some of the most interesting and informative data arose from discussions with, and observation of, public participants. First, were the reactions, unrestrained by formal experimental setting, with the most common being: clenching of the little fingers on both hands, rubbing the little finger on the illusioned hand, declaring “Witch-craft!,” counting “one, two, three, four, five, si … .what?,” laughter, disbelief, confusion, swearing (the children of today!), and, of course, describing it as “So weird.” Further interrogation of high and non-responders revealed two unusual but real experiences on the sensory integration spectrum. At one end of the spectrum, people reported “seeing” the extra finger. While most people only reported feeling a finger jutting out at the angle prescribed by the experimenter’s stroke on the table, this small subset (perhaps as few as 1/500) reported seeing an extra finger that promptly disappeared. The authors interpret this as an extreme weighting toward somatosensation over vision with the brain making sense of sensory input by filling in visual content. At the other end was a slightly larger subset that reported feeling only five touches on the hand instead of the delivered six. Unlike some, who identified two touches to the little finger (breaking the illusion), these people felt no touch at all when the experimenter’s finger was not seen to be in contact with the skin on the reflected hand, despite the experimenter’s finger firmly running down the full length of the unseen finger. The authors interpret this as representing an extreme weighting toward visual information over somatosensory input. What may also be interesting is that this particular phenomenon was observed in two female members of the same family, while the two males of that family both strongly reported feeling six fingers. It is noteworthy that a potential alternative outcome, that of perceiving a wider finger, was not reported. Following (mis)localization of the first touch, the more eccentric location of the second touch is congruent with the location of seen touch which was interpreted as a different, additional digit, although this may have been partly due to contextual framing.

This illusion exploits the somatotopic representation of touch to create a robust illusion of a supernumerary finger without postural manipulation or fake body parts and demonstrates the instantaneous remapping of touch onto an external frame of reference for the body through visuo-tactile integration.

## Supplementary Material

Supplementary material

## Supplemental Material

The supplemental material is available at http://IPE/sagepub.com/supplemental
