# Supplementary material for: The Anne Boleyn Illusion is a Six-Fingered Salute to Sensory Remapping
Source: Iperception. 2016 Sep 21;7(5):2041669516669732. doi: 10.1177/2041669516669732 (PMC5034331; doi:10.1177/2041669516669732)
Supplement: Supplementary material [file AnneBoleyn260516RNbR1.pdf]

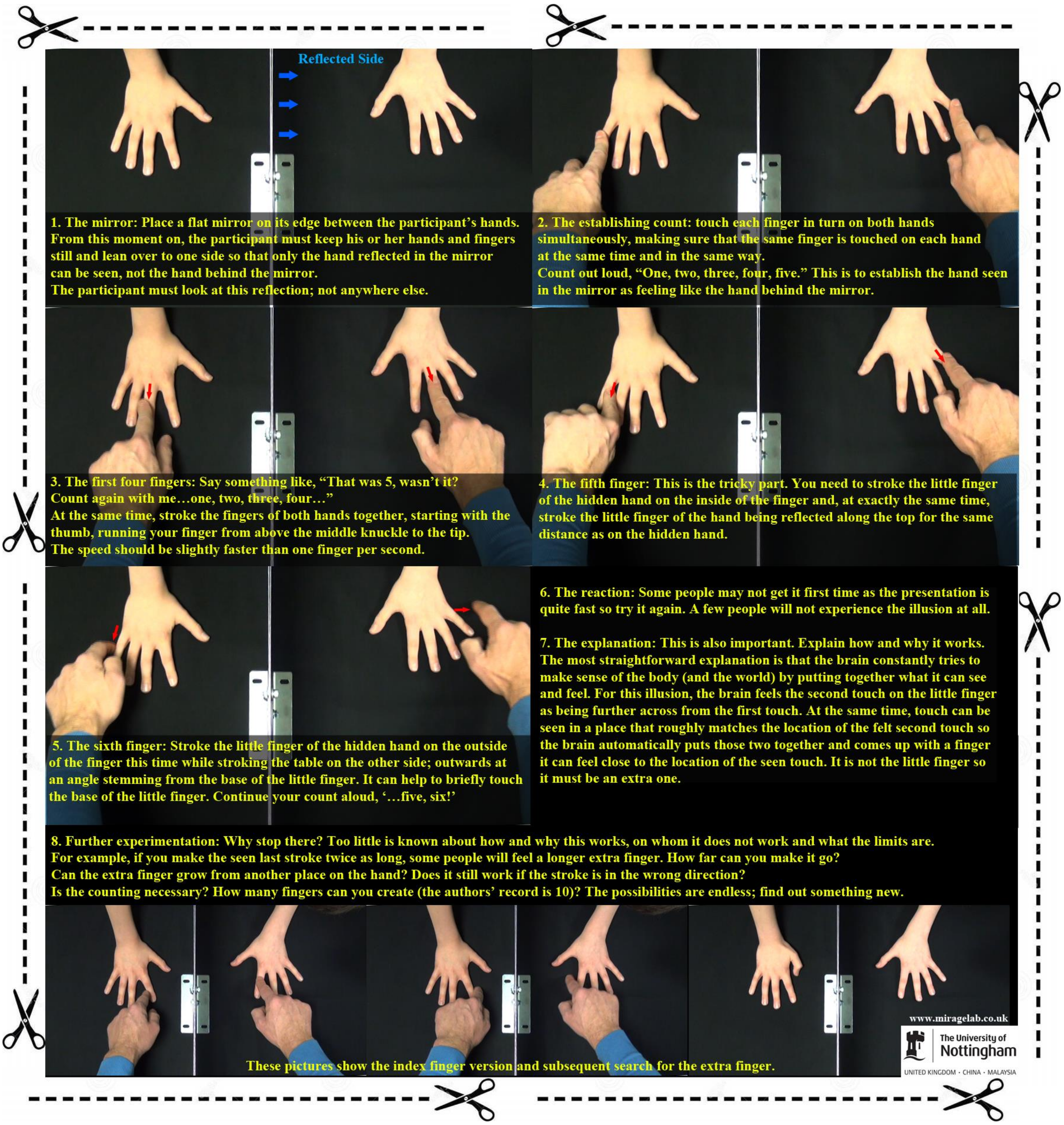

Supplemental Figure 1. Cut-out-and-keep instructions for conducting the Anne Boleyn Illusion.

Note: These instructions are intended for potential distribution to the public to enable accurate replication and to prompt further investigation in the home as well as in the laboratory.
